# A Case Series of Diverse Cardiac Abnormalities in Collegiate Athlete with COVID-19: Role for Multimodality Imaging

**DOI:** 10.1155/2022/3259978

**Published:** 2022-04-08

**Authors:** Vanessa-Rose G. Turpin, Kyle Goerl, Chen Hoe Chow, Carl J. Ade

**Affiliations:** ^1^Department of Kinesiology, College of Health and Human Sciences, Kansas State University, USA; ^2^Lafene Health Center, Kansas State University, USA; ^3^Cotton O'Neil Heart Center, Stormont Vail Health, USA; ^4^Physician Assistant Studies, College of Health and Human Sciences, Kansas State University, USA; ^5^Johnson Cancer Center, Kansas State University, USA

## Abstract

**Introduction:**

Since the COVID-19 pandemic there is concern for subclinical cardiac pathology in the absence of clinical symptoms in collegiate athletes, we present 4 cases of abnormal left ventricular global longitudinal strain (LVGLS), a “red-flag” for potential COVID-19 myocardial disease, following diagnosis with diverse abnormalities reported via multimodality imaging weeks into recovery.

**Methods:**

Cardiac imaging studies consisting of transthoracic echocardiography (TTE) and cardiovascular magnetic resonance imaging (CMR) were performed 10 days post-COVID-19 diagnosis and several weeks into recovery.

**Results:**

Initial TTE revealed abnormal left ventricular global longitudinal strain (LVGLS), an identified “red-flag” for potential COVID-19 myocardial disease. Further CMR imaging revealed potential recent/prior myocarditis in 1 athlete. Follow-up TTE several weeks later revealed a return to normal LVGLS. Conversely, 2 cases with normal CMR imaging had a LVGLS that remained abnormal >30 days into recovery.

**Conclusions:**

These individual cases highlight the substantial differences in echocardiographic and CMR abnormalities between athletes with confirmed COVID-19.

## 1. Introduction

With increased concerns for coronavirus disease-19- (COVID-19-) induced cardiac injury in athletes, numerous recommended screening procedures have been proposed; however, the differential pattern of cardiac abnormalities in individual athletes remains unknown [1]. Moreover, recent work suggests in the acute stage a presence of myocardial and pericardial inflammation in some athletes, but it is unknown if this persists into weeks of recovery. We describe a series of 4 competitive athletes with COVID-19 cardiac abnormalities. All patients provided written and verbal consent for this study which was approved by the Institutional Review Board of Kansas State University and conformed to the standards set forth by the Declaration of Helsinki.

## 2. Case 1

**Figure 1:**
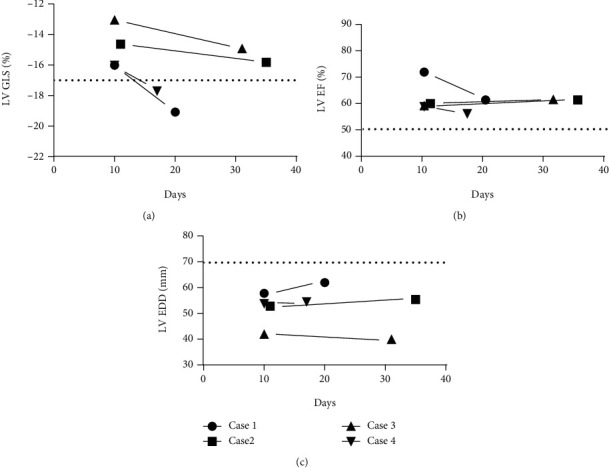
Transthoracic echocardiography findings following COVID-19 diagnosis. (a) LV GLS at ~10 days postinfection is abnormal in all cases [1]. (b) LVEF was normal in all cases. (c) LV dilation (LVEDD) [1] did not occur following infection.

An 18-year-old male collegiate athlete presented with fever, cough, and sinus congestion but denied chest pain and shortness of breath. The test for COVID-19 returned positive. Ten days into recovery, he had normal sinus rhythm, was normotensive, and had a normal 12-lead ECG. High-sensitivity troponin T (hsTnT) was normal (<0.010). Transthoracic echocardiography (TTE) showed mild left ventricular (LV) hypertrophy and cavity dimensions, suggestive of athletic remodeling. Mild dilations of right atria (RA) and left atria (LA) were noted with mild tricuspid valve regurgitation. LV ejection fraction (LVEF) was normal, but global longitudinal strain (GLS) was abnormal (-16%) with normal diastolic function (Figure 1). Cardiovascular magnetic resonance imaging (CMR) was performed 20 days into recovery, showing mild dilation of the LV and RV along with mitral and tricuspid valve regurgitation but no signs of myocarditis (no edema, T1 alterations, or Late Gadolinium Enhancement (LGE)). A repeat TTE 31 days into recovery showed improved GLS (-19.1%) and normal LVEF.

## 3. Case 2

A 19-year-old male collegiate athlete tested positive for COVID-19, and 11 days into his recovery, there were no indications of arrhythmias or murmurs with normal hsTnT (<0.010). Contemporaneous TTE revealed a normal camber dimensions, LVEF, and diastolic function, but abnormal GLS (-14.6%) with mitral and tricuspid valve regurgitation was observed. CMR performed 19 days into recovery revealed no signs of myocarditis (no edema, T1 alterations, or Late Gadolinium Enhancement (LGE)). A repeat TTE 35 days into recovery showed a still abnormal but improved GLS of -15.8%, with normal LVEF%.

## 4. Case 3

A 20-year-old male collegiate athlete presented with symptoms of a headache, nausea, difficulty breathing, sore throat, and fatigue after testing positive for COVID-19 but denied any symptoms of chest pain or shortness of breath (SOB), and hsTnT was normal (<0.010). TTE was performed 10 days following diagnosis, and athlete reported continuing symptoms of a headache, stuffy nose, and fatigue. He was hypertensive (130/58 mmHg) but had a normal 12-lead ECG and LVEF. TTE showed mild dilation of the RV and RA and abnormal LV GLS of -13%. CMR was performed 22 days into recovery showing no signs of myocarditis (no edema, T1 alterations, or Late Gadolinium Enhancement (LGE)) and normal myocardial perfusion. Follow-up TTE, 31 days into recovery, revealed an abnormal but improved GLS (-14.9%).

## 5. Case 4

**Figure 2:**
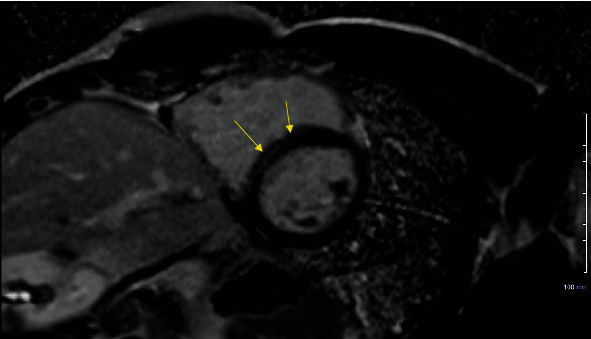
Typical CMR in case 4 of LGE-indicated recent/prior myocarditis.

An 18-year-old male collegiate diagnosed with COVID-19 reported a mild cough during infection but denied chest pain, SOB, or fever. Ten days into recovery, he had a normal ECG and hsTnT (<0.010). TTE revealed an abnormal LVGLS of -16%, but normal LVEF and diastolic function. CMR performed 15 days into recovery showed an area of midmyocardial LGE, indicating potential recent/prior myocarditis, with normal LV wall motion and LVEF (Figure 2). TTE was performed 2 days later and showed an improved and normal LVGLS of -17.7%.

## 6. Discussion

Early reports have indicated that SARS-CoV-2 infection elicits cardiac injury in up to 2 out of 5 hospitalized COVID-19 patients [2–9], with elevated cardiac troponin I, a marker of acute myocardial injury, within 24 hrs of hospital admissions associated with death (hazard ratio 3.23 and 95% CI 2.59-4.02) [2]. Moreover, data from China has revealed that mortality rate was higher among patients with vs. without cardiac injury, even after adjusting for age and prior comorbidities, with COVID-19-related cardiac injury associated a 5-fold increase in required ventilation [4, 6, 9–12]. Recent data from Puntmann et al. has also suggested that in middle-aged adults recently recovered from COVID-19, of which 33% required hospitalization, a majority of patients had cardiovascular involvement, as detected by CMR, with 60% exhibiting ongoing myocardial inflammation [13].

Unfortunately, to date, most of our knowledge on COVD-19-induced cardiovascular complications is limited to hospitalized patients, with a paucity of information on the use of imaging modalities for diagnosis and follow-up of myocardial involvement in younger nonhospitalized individuals. Recently, Joy et al. evaluated cardiac function in health-care workers (mean age 37 years) 6 months following COVID-19, of which 85% were mildly symptomatic and 15% asymptomatic at the time of diagnosis [14]. Their measurements of cardiac involvement via CMR scanning revealed no difference in cardiac structure, function, or tissue characterization between recovered COVID-19 individuals and matched controls. While CMR imaging provides valuable insight into cardiovascular involvement with COVID-19, it is not readily available in all clinical settings, with TTE more commonly used as the method for early cardiac screening following infection.

Assessment of COVID-19-induced cardiac abnormalities, against the background of athletic remodeling, presents a particularly unique challenge in identifying individuals at risk for pathologic outcomes following infection. In addition, most athletic departments do not have the capabilities or capacity for onsite cardiac evaluation following COVID-19. When follow-up evaluation for cardiac involvement is required, this challenge is further exacerbated when different imaging modalities are utilized. Thus, there is a critical need to improve our understanding on the differential pattern of cardiac abnormalities that may exist in this unique population. Here, we present 4 cases of abnormal LVGLS, an identified “red-flag” for potential COVID-19 myocardial disease [1]. CMR revealed potential recent/prior myocarditis in 1 case, but a return to normal LVGLS. Two cases had normal CMR outcomes but LVGLS remained abnormal >30 days into recovery. This highlights the substantial variability in echocardiographic and CMR abnormalities between athletes with confirmed COVID-19. Recommendations for screening of athletes with COVID-19 include the use of hsTnT and echocardiographic assessment of chamber size, wall motion, and systolic and diastolic function, with subsequent CMR recommended when indicated [1]. We demonstrate variability in multimodality imaging in the characterization of potential COVID-19 cardiac injury in collegiate athletes, which highlights the challenges of managing COVID-19 and determining the appropriate workup in this population. At the individual patient level, an abnormal LVGLS and the temporal characteristics into recovery do not appear to be predictive of CMR-indicated myocarditis.

To date, there are a limited number of studies investigating the potential cardiac consequences of COVID-19 in the athletic populations, with fewer extending into recovery (Table 1). In a study of 22 COVID-19-positive athletes, all had normal troponin and LVGLS [15], with one exhibiting meeting CMR criteria for suggested myocarditis. In agreement, a recent study by Rajpal et al. looked at 26 COVID-19-positive student athletes and reported that no athlete had markers of elevated troponin, but 46% showed the presence of CMR-indicated LGE, further highlighting how variable CMR data is between studies and the potential for myocarditis in the absence of elevated biomarkers [16]. Others have reported ~40% of COVID-19 athletes exhibit late pericardial enhancement and with more than one-half of athletes showing subclinical myocardial and pericardial disease [17]. Additional work in professional athletes with COVID-19 revealed acute cardiac injury in 2.5% with CMR-confirmed inflammatory heart disease in 18.5%, myocarditis in 11.1%, and pericarditis in 7.4% [18].

To date, both direct and indirect effects of the SARS-CoV-2 virus on cardiovascular outcomes have been postulated but remain incompletely understood [19–22], thus limiting clinical decision-making regarding patient triage and treatment. Recent work by Greulich and Klingel suggests a very heterogenous presentation of myocardial inflammation in patients with a history of COVID-19 following endomyocardial biopsy, further highlighting the unknown pathogenesis of COVID-19-related cardiac inflammation [23]. Moreover, while CMR and other imaging modalities, like that used in the present study and previous work provide valuable prognostic insight, they often provide different pathological information relative to endomyocardial biopsy further complicating our understanding of the underlying mechanisms mediating COVID-19 myocardial injury [24]. Thus, this previous work, coupled with the divergent response reported in the present cases, highlights the challenges associated with the clinical evaluation of cardiac abnormalities in athletes following COVID-19 diagnosis.

The diverse temporal responses of our cases highlight our limited understanding of the time course of LV function changes as they relate to CMR parameters. Taken altogether, the consequences of COVID-19 infection still remain unclear and future research looking into the long-term effects of this disease is warranted, with clear indication that no screening modality provides a complete picture of potential cardiac abnormalities.

## Figures and Tables

**Table 1:** Recent reports on cardiac abnormalities in athletes with COVID-19.

First author (year)	No. of participants	Average age ± SD (years)	No. of asymptomatic (%)	No. of symptomatic (%)	Elevated troponin I or T levels	LGE presence	No. of myocarditis
Rajpal et al. (2020) [16]	26	19.5 ± 1.5	14/26 (54%)	12/26 (46%)	0/26	12/26	4
Vago et al. (2020) [25]	12	23	2/12 (17%)	10/12 (83%)	0/12	0/12	0
Brito et al. (2020) [17]	54	19	16/54 (30%)	38/54 (70%)	1/54	1/54	0
Clark et al. (2020) [15]	22	20	5/22 (23%)	17/22 (77%)	0/22	2/22	1

Each study analyzed serum troponin I or T levels and utilized CMR to evaluate LGE and other cardiac parameters. Only Clark et al. [15] performed transthoracic echocardiography in addition to hsTnT and CMR.

## Data Availability

All data used in this case report is readily available through cited literature or is protected patient information, which cannot be released.
